# Arterial Degeneration

**Published:** 1901-06-29

**Authors:** 


### ARTERIAL DEGENERATION.

Dr. Syers[1] draws attention to the importance of studying the condition of the arteries if one would gain a full and accurate conception of the life prospects of an individual. Of course we all recognise the frequency with which the aorta is atheromatous and the cerebral vessels are degenerate in connection with renal disease, but it is by no means unusual to meet with cases in which the same condition of vessels is present without any cardiac or renal disease whatever, and it is to this primary arterial degeneration that he wishes to draw attention. This vascular change may occur in those who are far below the age at which ordinary atheromatous processes are generally thought to become common, and may be met with in persons who have never suffered from syphilis or from the effects of alcoholic excess. It is a very interesting question how far the healthy state of the arterial system is due to mode of life and personal surroundings. Dr. Syers holds that the environment has much less to do with the matter than is often thought, the chief factor in causing the retrograde change being heredity. A father, himself the easy victim of atheroma, is in the highest degree likely to transmit the same tendency to his son, while in long-lived families a tendency to sound and elastic arteries is transmitted from father to son for many generations. One can, for example, hardly avoid observing how often really aged people, say those over ninety, are provided with good and comparatively undegenerate arteries. Thus looked at from one point of view, arterial degeneration can scarcely be regarded as a diseased process, but only as a natural involution by which "this mortal coil" is shuffled off. It becomes a disease only when it occurs before its due time. It is curious to notice the unity and sameness of the morbid process which affects all organs in old age; everywhere there is a tendency to fibroid transformation. The heart, the lungs, the liver, the kidneys, all show the same change, indeed, in the latter, disease merely anticipates the pro-

[1] Treatment, June.

cess of natural dissolution. If the kidneys of a very aged person are compared with some very chronic forms of Bright's disease occurring in a young child, it may not be easy to discriminate between the two organs. Thus the reversion of all structures, however complicated, to the final form of fibrous tissue, would seem to be nature's method of bringing life to an end, and when this process affects the vessels, it is one of the most speedy means of causing death. It is important to observe the curious limitation of the atheromatous change to certain portions of the aorta, or to certain vessels, and it is worth bearing in mind that this partial distribution of arterial change is more likely to be met with in those cases in which it is a primary, substantive, independent disease of the vessels, and not a mere accompaniment of kidney or cardiac degeneration. In investigating the condition of the arterial system as regards the presence or absence of atheroma, it is to be remembered then that it is not enough merely to ascertain the condition of the radial artery, for the brachial or the temporal arteries may show signs of atheroma when the radial is all right, or there may be a systolic murmur at the base of the heart, showing atheroma of the aorta; that arterial degeneration is in a very large number of cases is due to an inherited vice often connected with gout, and is very often indeed that weak point in the patient's constitution which determines his death at a comparatively early age; that this being so, all that can be done is by care and watchfulness to postpone the onset of the malady as long as possible; that only in cases in which the disease is induced by syphilis or drink can treatment be expected to effect any great improvement; and that much more attention than is customary should be devoted to the investigation of the special liability of any particular case to this condition, as the latent tendency to this vascular change is not only very widespread but is fraught with the greatest risk to premature death.

